# Good care and adverse effects: Exploring the use of social alarms in care for older people in Sweden

**DOI:** 10.1177/13634593231185260

**Published:** 2023-06-30

**Authors:** Doris Lydahl

**Affiliations:** University of Gothenburg, Sweden

**Keywords:** ethnography, technology in healthcare, theory

## Abstract

In Nordic countries, ‘welfare technology’ is a concept used increasingly by policymakers when discussing the promise of digitalisation in care for older people. In this paper, I draw on data from 14 qualitative ethnographic interviews with employees in municipal eldercare in Sweden, as well as observations carried out at a nursing home, to suggest the importance of studying how good care is enacted through welfare technology, whilst simultaneously attending to the adverse effects sometimes consequent from these practices. In this article, I explore what values are supported when doing care with welfare technology, and what values are neglected in this process. The theoretical starting point for this article takes its inspiration from recent discussions of care within Science and Technology Studies (STS). Employing a *double vision of care*, the article argues for the importance of understanding how good care is enacted with technology, while also attending to what these care practices exclude and neglect. Focusing on the use of *social alarms*, the article shows that when doing care with such technology, values such as independence, safety and some forms of togetherness and availability were enhanced; while other values such as other forms togetherness and availability, a stress-free working environment and functionality were neglected.

## Introduction

In Sweden, digital technologies used in care for older people are increasingly discussed as ‘welfare technologies’. Welfare technology is a concept only used in Nordic countries and has been described as a ‘new mantra for reforming Nordic public health and social care’ (Kamp et al., 2019: 1). National and local political strategies of welfare technology emphasise that technologies will make it possible for older people to ‘live a self-reliant and independent daily life, and reduce their dependency on public services and benefits’ (Dahler, 2018: 2). The concept welfare technology itself is very broad and there is no consensus on how to define it in the Nordic countries. In 2015, the Swedish National Board of Health and Welfare published a definition of welfare technology as ‘digital technology aiming to contribute positively to the welfare of the population by increasing safety, activity, participation and independence for people who have, or risk developing, a disability’ (Socialstyrelsen, 2015, author’s translation). Examples of welfare technologies mentioned in this definition are door sensors, social alarms, technologies for video-communication and robot pets. Moreover, welfare technology is seen as a resource to tackle strained budgets, changing demographics and staffing shortages in Sweden and beyond (Frennert and Baudin, 2021; Nilsson et al., 2022).

Research on welfare technology in elder care is a small but growing field. Studies have explored how older people use, perceive and experience digital and welfare technology (Frennert et al., 2012, 2013). Åkerlind (2017) concludes that welfare technology can provide safety and increase participation if used correctly. With a specific focus on communication and safety, Åkerlind (2017) argues that participants might be able to attain a feeling of togetherness and affection through communication via welfare technology. In contrast, others warn that core values of care are being lost when introducing digital welfare technologies (Frennert, 2019). A few studies attend to care workers’ attitudes and views on the use of welfare technology. Nilsen et al. (2016) argue that resistance to welfare technology ‘may play a productive role when the implementation is organised as a co-creation process’ (p. 1). Hasselblad (2021) concludes that actors involved in the design, implementation and use of welfare technologies have different views and perspectives influenced by their respective professional norms and knowledge traditions. Some studies focus on the values of welfare technologies. Grosen and Hansen (2021) argue that in the policy setting work surrounding welfare technologies for older people in person-centred care, privacy and security are important guiding values needing to be negotiated. Building on an understanding of care ‘as realized in heterogeneous collectives of human and nonhuman actors’ (Grosen & Hansen, 2021: 1) the article illustrates how new care practices, established in relation to the introduction of welfare technology, led to value-conflicts, especially between the values of privacy, on the one hand, and security on the other. The handling of the tensions caused by the norms in friction lead to new insecurities, work intensification and to ethical dilemmas and paradoxes for care workers.

Research also indicates that the use of such technology can produce stress for care workers. Bergschöld (2018) uses the term ‘time labour’ to describe how care workers are changing the way they work in response to time pressure, cutting home visits as short as possible and ‘using technology to strategically produce and maintain quantitative representations of themselves as skilled workers’ (p.3). Therefore, rather than leading to a reduction of workload, research shows that the implementation welfare technology itself requires work (Ertner, 2019; Grünenberg et al., 2022; Nickelsen, 2013; Tøndel and Seibt, 2019). Similarly, research shows that the introduction of welfare technology will likely lead to new work processes and new working conditions for care workers (Frennert, 2019). It is often just the visible aspects of care work that get regarded as official work, as authorised and documented. However, the work performed to manage new tasks in relation to welfare technology is often invisible (Ertner, 2019), as it is related to more mundane inventiveness, tinkering and maintenance of such technologies (Lydahl, 2017, 2021a).

Many hopes and expectations are put on welfare technology to provide good care. However, as argued by Mol et al. (2010), the good and bad ‘tend to be intertwined’, and good practices may also have some negative side-effects (p. 13). In short, welfare technology is a double-edged sword. In this article, I aim to describe ways of doing good care with welfare technology while also attending to the adverse effects that sometimes come with these care practices. Seeing values and care as enacted in practice (Pols, 2008) I ask what is valued when doing care with welfare technology and what other values are neglected in this process. To do this I focus on the most used welfare technology in Sweden – the social alarm system (Socialstyrelsen, 2020). This is a technology installed either in a user’s home, or nursing homes, that makes it possible for the user to call for help in urgent situations or if there is a problem.^1^ My sample group will be a medium-sized municipality in western Sweden.

The increased interest in social alarms should be seen against the reforms which Swedish eldercare underwent in the 1960s. During this period, ideals such as normalisation and autonomy came to be valued more, and it was suggested that older people should have access to care and services in their ordinary homes instead of simply in institutional care homes. In a report published in 1976 the National Board of Health and Welfare and the Swedish Agency argued that social alarms increased the prospects for older persons to continue living at home with access to care and services just a press of a button away (Schmidt, 1986). During the early 1980s the introduction of personal alarms proliferated in Swedish municipalities. Today, digital social alarm systems are the most common welfare technology in Sweden (Socialstyrelsen, 2020). They are used both in home care services and in nursing homes. Social alarms are prescribed to their users by the person in charge of their case at the social service office, regardless of whether the user is living at home or in a nursing home. It was not until recently that social alarms were digitalised. In 2014, only 14% of all social alarms were digital, while the rest were still analogue and connected to the telephone network (Socialstyrelsen, 2020: 20). Most social alarm systems contain similar components and usually consist of three parts: an alarm button which usually looks like a clock or a pendant; a base unit for digitally transferring the alarm signal; and alarm phones, or a specific app on a regular phone, for receiving alarms (Chang, 2021). How the alarm systems are governed, however, differs across the various municipalities. In some municipalities, the alarm systems are governed ‘in-house’, but in many cases they are managed by an outside operator (Socialstyrelsen, 2020).

In the following section, I will introduce the theoretical framework grounding this article. I will discuss how to understand good care, and how to make sense of the bad it may contain. Next, I shall outline the experimental design that was used, the empirical data the article builds on and how it was collected and analysed. I will then introduce my findings, showing that while good care can indeed be made with social alarms, this care is *selective* and while supporting certain values such care also neglects other values. Finally, I will discuss what we can learn from taking such a double vision approach to welfare technology enacted care.

## Understanding technologies of care and care practices: A theoretical framework

Focusing on both the good and potential adverse aspects of welfare technology enacted care involves a *double vision of care* (Lindén and Lydahl, 2021). This double vision prescribes that care should be understood in terms of its specificity and context, while also emphasising the importance of critically interrogating care practices.

The double vision of care is based on a combination of a *care-in-practice-approach* (Mol et al., 2010) as well as a *critical care approach* (Martin et al., 2015) which have been developed in feminist science and technology studies (STS). Both these approaches agree that care is a doing, a practice. This focus on practices is common within STS studies. Material semiotics draws attention to ‘the messy practices of relationality and materiality of the world’ (Law, 2009: 142). As such, in care studies, what is in focus is not human individuals or their properties, but rather the actions and practices of humans and non-humans (Siira, 2022). Care scholar Maria Puig de la Bellacasa describes care as a ‘a manifold range of doings needed to create, hold together and sustain life and continue its diverseness’ (Puig de la Bellacasa, 2017: 70).

Drawing on an empirical philosophy tradition, the care-practice-approach stresses that care is not something to be judged ‘in general terms and from the outside, but something to do, in practice’ (Mol et al., 2010: 13). The approach prescribes that ‘good care’ should be understood as ‘persistent tinkering in a world full of ambivalences and shifting tensions’ (Mol et al., 2010: 14), and that care is about being attentive, that is, ‘paying attention to the particularities of the situation of the people being cared for’ (Ceci et al., 2013: 11). In this approach, care is conceptualised as being constituted by collectives which also include technologies (Mol et al., 2010). Therefore, care is seen as heterogeneous and materially-embedded. Technologies are also seen as a part of care (Lydahl, 2021a; Van Hout et al., 2015). Hence, for example, a relationship between a human and a robot dog can be caring and emotionally significant, and technologies which are meant to educate and monitor patients can create affection and interaction (Pols and Moser, 2009). It is essential to note, however, that technologies do not perform care on their own but instead become ‘part of new forms of caring relations and activities’ (Mort et al., 2015: 444).

A key issue in the critical care approach is to draw attention to questions of power and to the exclusions reproduced in and through care (Martin et al., 2015). Critical care scholars have demonstrated how care is a ‘selective mode of attention’ (Martin et al., 2015: 627) which, in the process of valuing some things, necessarily excludes others. Thus, it has been suggested that care should not be conflated with affection and positive feelings. It is important to acknowledge and critique the violence sometimes committed in the name of care and that care, therefore, has a ‘dark side’ (Martin et al., 2015: 627).

A double vision approach to analysing care, therefore, provides a good framework for both understanding care made with welfare technology, as well as its adverse effects. Furthermore, this perspective on care is based on the idea that ethical theory is situated – it is located and grounded in practice, rather than guiding our actions from outside (Lindén & Lydahl, 2021). Pols (2008) suggests that the location of ethical theory in practice can help the researcher to interfere ‘in the practices studied by opening implicit notions of good care for (self) reflection’ (Pols, 2008: 52). By focusing on the practice of doing care with welfare technology, this article seeks to provide situated and critical knowledge and open up practice-grounded ethical reflections.

## Methodology

### Design

I draw on a combination of qualitative approaches in this study and build on a dataset derived from ethnographically driven interviews and mobile ethnography to scrutinise practices of doing care with social alarms for older people in Sweden.

### Study context: Nursing homes in Sweden

One of the aims of eldercare in Sweden is to help older people, and people with disabilities, to live an independent life (Agosti et al., 2015). Homecare services are used to reach this goal, so older people are able to live in their own homes for as long as possible. Homecare services in Sweden generally include help with domestic tasks like cleaning and cooking, as well as personal care such as bathing and getting dressed. Medical tasks can be included, for example distribution of medication and treatment of wounds. Additionally, emotional and social support are regarded important aspects of the service (Szebehely and Trydegård, 2012).

In Sweden, persons who can no longer manage their everyday life in their own home, after assessment has been conducted, are offered a place to live in nursing homes with 24-hour staff. Eligibility criteria for being admitted into a nursing home often include cognitive limitations such as dementia, or severe illnesses and/or functional limitations. Nursing homes are usually large buildings that include smaller units composed of apartments and communal areas (Johansson et al., 2020). At the nursing home, the resident – as the elderly persons are usually called – can get round-the-clock care and service like those included homecare services but also including the serving of three cooked meals a day, plus snacks, as well as on situ medical care from a registered nurse or general practitioner. Both homecare services and nursing homes can be provided by private actors yet still be funded by the municipality.

The staff performing homecare services and working in nursing homes in Sweden are assistant and registered nurses. Assistant nurses are mainly responsible for providing basic care and domestic work tasks, while registered nurses with a three-year university education in nursing are responsible for the medical care. Most staff are assistant nurses, who are either licensed practical nurses or nurse’s aides. Licensed practical nurses have three years upper secondary education focused on care and social services, while nurse’s aides have limited or no education in caring. In this article I refer to assistant nurses simply as nurses.

### Procedure and data collection

I draw on empirical data collected in a medium sized municipality in the western part of Sweden. The municipality is relatively prosperous, with low unemployment rates and overall good socioeconomic conditions. In comparison to other same sized municipalities relatively fewer people are foreign born.

Between September 2020 and June 2021, I carried out 14 interviews with staff in the municipal eldercare. Twelve out of the 14 interviewees were female, and all interviewees were of Swedish origin. Due to COVID-19 all interviews were conducted via videocall over the phone. Interviews lasted between 29 and 67 minutes and were transcribed verbatim. I interviewed managers and administrative staff with responsibilities related to welfare technology, and assistant nurses. Interview participants were recruited through managers. To achieve a broad representation, they were asked to recruit participants who were both older and younger, and who were more and less familiar working with welfare technology.

The interviews draw from Sarah Pink’s (2015) notion of ethnographic interviewing. Herein, people discuss their ‘lives, beliefs, values, opinions, experiences, practices and more in a focused way’ (Pink, 2015: 80). The approach focuses on the mundane, practical and the everyday. To this end, I asked the interviewees to talk about their everyday work, what a normal day at work usually looks like, what type of welfare technology they encounter in their everyday work, and what routines they have. This opened up the interview so I could ask follow-up questions about ethics and values.

In addition, I have carried out 24 hours of mobile ethnographic observations, also known as shadowing (Lydahl et al., 2021; Czarniawska, 2007), at a nursing home in the municipality. Shadowing is a practical approach for studying practices of care (Lydahl, 2021b) – it involves following ‘selected people in their everyday occupations for a time’ (Czarniawska, 2007: 17) and it can be particularly valuable as it enables the researcher to ‘learn what is going on, rather than what should be going on, as resulting from formal documents and even interviews’ (Czarniawska, 2007: 33). During my shadowing, I followed six assistant nurses for parts, or the whole, of their work shift. All the nurses I followed were female and of Swedish origin. While in the field, I took continuous handwritten field notes, which were transcribed into thick descriptions, typed into the computer, as soon as possible after the shadowing was completed.

All participants gave their informed consent to participating in the study. Pseudonyms were used throughout the reporting of the findings, and any identifying characteristics were anonymised. The study was reviewed and approved by the Swedish Ethical Review Authority (Dnr 2020-01344).

### Analysis

Analysis of the data involved three steps, drawing on Tavory and Timmermans (2014) abductive approach. The first step is *mnemonic*, which includes listening, reading, re-reading and noting initial ideas. During this step, care as a theoretical approach was important, as the abductive approach prescribes an extensive familiarity with existing scholarship from the outset. Drawing specifically on a care in practice approach, I coded all instances that were expressive of or practical examples of, ideals r’egarding good care and the social alarm (Author). I also coded all instances of practical doings related to the social alarm and who was doing what. Axial coding followed the initial coding, in which I compared codes across the entire dataset and merged similar codes (Tavory and Timmermans 2014: 54). The second step is *defamiliarisation* which involves multiple re-readings of material and ‘slow’ examination of the data to identify empirical puzzles (Tavory and Timmermans, 2014). To facilitate this process, I read the material ‘against the grain’ (Lindén and Singleton, 2021) and made use of *disconcertment* (Verran, 2001) as heuristics. Both these heuristics emphasise the importance of stepping out of dominant narratives, readings or interpretations. Disconcertment can be described as an unsettling feeling of ‘seeing certainty disrupted’ (Verran, 1999: 141). Its disruptive qualities have been shown to open possibilities for thinking in ways that diverge from dominant narratives. Here the researcher’s intellectual, affective or bodily irritation or feeling is used as a tool to search and further specify and relate to their research concerns. Making use of disconcertment as a heuristic for this article, I have focused on instances where I during fieldwork felt unease in field, to stimulate readings against the grain. Reading the material against the grain and making use of disconcertment made it possible for me to focus on the adverse effects of doing care with welfare technology, as adverse effects often do not match the dominant narrative. In this way, reading against the grain and disconcertments have provided a way for me to employ the double vision of care as it has helped me to focus both on the actual practices of care and on its darker sides. The third step in the abductive method is *revisiting observations* which includes re-reading and re-coding the material, changing, modifying and regrouping initial interpretations based on lessons learned from the defamiliarisation.

## Findings

### Eating breakfast alone

Residents living in the nursing home where I did observations usually have little independence with their days being highly structured around a schedule set up to cater for the needs of everyone living in the home. For example, residents cannot decide exactly when they want to eat breakfast, as breakfast is served at nine every morning. In the field, I noticed that nurses often were attentive to situations where the independence of the residents could be promoted. Independence was often mentioned as a good which could be protected and sustained through the social alarm. Independence is indeed a fêted ideal in Western eldercare (Barken, 2019; Purkis 2012). This is so, especially in homecare where the aging-in-place policy is dominant. Independence is a key value to be protected (Wiles et al., 2012), and nursing homes are regularly associated with a loss of independence and increased dependency (Stabell et al., 2004). In Sweden, the introduction of welfare technology is precisely aimed at increasing the independence of people who are, or risk becoming, disabled both at home and in nursing homes. Nilsson et al. (2022) argue that independence is normative in Swedish health and social care policy concerning digital technologies.

Nurses talked about the ways in which the social alarm helped residents to maintain their autonomy and independence. For example, rather than deciding *for* the residents when they should dress, take their medication or take a shower, the nurses argued that residents could use the social alarm to tell the nurses when they needed something. This can be illustrated by a situation at the nursing home involving the resident Hans and the nurse Linda.

*Hans is a younger resident with maintained cognitive functions living in a two-room apartment at the nursing home. He is confined to an automated wheelchair and spends most days in his room where he is regularly visited by his friends and family. Linda gives Hans his medication while asking him if he wants to get dressed before or after she brings him his breakfast. Hans is unsure and after some discussions they decide that Hans will call Linda on the social alarm when he is ready for breakfast and for getting dressed. An hour or so later, just after 9am, Linda and I sit in the residents’ dinner room. Linda’s mobile phone starts beeping and she tells me that she can see that it is Hans calling through the social alarm. She tries answering him through the application, but it won’t work. Her phone beeps again and this time she succeeds in answering the call. After finishing the call, she tells me that Hans wants to get dressed and to get some breakfast to his room.*

I interpret this interaction as showing Linda trying to find a way to make Hans’ morning as comfortable and pleasant as possible. She gives him different options for when he wants to get dressed and eat breakfast, trying to make sure that Hans is independent, albeit confined to his wheelchair. Rather than forcing the schedule of the nursing home on him, she tries to find a way of adapting the routines to him.

According to Mol et al. (2010) care is situated – it is not something which can be judged based on principles as if from the outside. Care is something done in practice, and is about finding ‘local solutions to specific problems [that] need to be worked out’ (Mol et al., 2010: 13). From this perspective, the above situation could very well be described as an example of good care made with technology. Linda is paying due attention to the *particularities of Hans’s situation* (Ceci et al., 2013). She considers his abilities, his wishes and needs – that he is well able to use the social alarm and that he is a bit unsure about when he wishes to get dressed and have breakfast. Rather than insisting that Hans must eat breakfast and get dressed at a pre-set time, Linda comes up with a *local solution* which fits Hans and his situation (Mol et al., 2010). This solution is that Hans will call on Linda when he is ready. Given that Linda did not ask Hans to come to the dinner room to eat breakfast with the other residents, this seems to be a routine procedure for Hans. When I ask Linda about this, she says that she and her colleagues try to be flexible with Hans’s schedule so that he can keep his independence and set up his days with visits from friends and family as he likes. To do so, the social alarm is key. The technology does not just help Linda and her colleagues to be attentive to Hans’ wishes. It also offers Hans a way of connecting with Linda and the other nurses. Thus, in this instance, care is given with and through technology, and independence as the outcome of care and the social alarm are interlinked. Indeed, one can argue that for Hans to keep some level of independence, he must learn to become *dependent* on, and entangled with, technologies such as the social alarm – though also on the care work of nurses such as Linda (cf. Pasveer et al., 2020).

*. . .and dinner together*

The story does not end here.

*Carol, a nurse who usually works at another unit, goes to pick up the residents that have not yet come out in the dining room. She picks up Hans in his wheelchair. In the dining room she asks a colleague at what table Hans sits and is informed that Hans usually calls on the social alarm and eats in his room. Carol answers that she is not familiar with the routines at the unit, but that she asked Hans if he wanted to join her in going to the dinner room and that he gladly agreed. Amina, who works in the kitchen, serves the residents their dinner. Today they are served fish cakes with sauce, potatoes, and vegetables. It smells good and my stomach grumbles. Amina asks how many fish cakes and potatoes the residents want and if there is something they do not want. Hans is in a good mood and talks with the other residents, the staff and me. He eats with a good appetite and asks for a second serving of fish cakes.*

When Carol first showed up with Hans, I felt a sense of unease. Why did she not know about the routines at the unit, I thought. Why did she not offer to let Hans eat dinner in his room as the other nurses usually do? Did this decrease the independence of Hans by interfering with his private schedule?

I returned again and again to the situation and re-read my fieldnotes. Carol’s action was a disruption from the routine of letting residents with higher cognitive functions call on the staff, for example, when they wished to be served dinner. Instead, she made Hans come out into the dining hall, like the other residents. Back in my office, I found that I had slipped into making assumptions based on the dominant narrative (c.f. Jerak-Zuiderent, 2015) – holding sway at the unit, and in Swedish eldercare policy at large – the assumption being that social alarms are a solution to a problem of a lack of independence and that independence is a value to be safeguarded. Taking my own uneasiness seriously and nurturing the *disconcertment* (Jerak-Zuiderent, 2015; Verran, 2001) which I felt when Carol picked up Hans, another potential narrative started to unfold. Thinking about the dinner situation, we can see that one possible adverse effect of the social alarm is that residents *might routinely become excluded from social events at the nursing home*. As critical care scholars Martin et al. (2015) argue, care necessarily excludes some things in the process of attending to others. Reading the first field-note *in light of* the second, what stands out is that Linda never asked Hans if he wanted to have breakfast in the dining room with the other residents. Therefore, an alternative narrative could be construed to suggest that, while good care can indeed be made with social alarms, this care is selective and *in the process of valuing independence it also neglected other values such togetherness.* Here, togetherness is about eating together and sharing a meal with others (though it can also mean other things, as we shall see in following examples).

This alternative narrative also suggests that a very specific vision of independence is envisioned in doing care with the social alarm. Independence, in this vision is about being free to pick and choose from the activities and to be able to be social outside the realm of the nursing home. Yet, independence can be conceptualised in other ways. Stabell et al. (2004) note, for example, that ‘mealtime appears to be a good opportunity to foster the independence of the residents’ (p. 677). Here independence is about being able to eat by oneself rather than being fed, and that it is done socially is seen as a much-valued bonus.

### Staff being a push of a button away

The nursing home where I conducted my observations consists of four units, composed of apartments and communal areas situated along long corridors. Though each unit is small, it is sometimes still difficult to find a nurse. When a nurse visits a resident’s apartment, they close the door, as a visit often includes changing a diaper, helping a resident to the toilet or performing other intimate tasks belonging to the private sphere (Twigg, 2000). During my observations, it became apparent that the social alarms played a significant role in providing contact with nurses when help was needed. The social alarm app in the nurses’ mobile phones beeped constantly. Residents called for the nurses when they need to use the bathroom, need help to get dressed, want to get down to the communal patio and when they want a cup of coffee – all things which are difficult or impossible for the residents to do by themselves. Residents also called for the nurses in more serious situations demanding immediate attention, for example, when they had fallen, were in pain or were sad.

In interviews with the staff, several said that being able to call for help was what the social alarm meant for the practical and everyday life of nurses and residents alike. One nurse talked about this poignantly:

*It is about safety for the residents. They can push a button and then we come. That’s a great safety measure for them. I must say that I think it is a great device, that they can push a button and we can talk with them through the speaker or visit them straight away. I think it is amazing in its simplicity.*

*[—]*

*If they push the alarm-button we can talk. . . they hear a voice in the speaker. We have heard you. We will be with you in just a moment. It’s a great feeling of safety for them.*

*IP 22, female nurse*

By this nurse’s account, the alarm makes the residents feel safe. This also makes her feel safe – she can care for the resident because it helps her doing what she needs to do when the residents need it: ‘It’s a great device’.

The type of care this nurse talks about is materially embedded. Nurses are made available *through* the social alarm and the speaker – the residents ‘push a button’ and the nurse answers. Availability is made easier through the social alarm. Instead of looking for a nurse, the residents have a way of connecting with the nurses ‘straight away’, and they are heard and responded to at once. Good care, according to these nurses, and from what I had seen in my observation, involves them being *available* for residents which increases their sense of *safety*.^2^ Nilsson et al. (2022) have described that e-health policies in Sweden place high expectations on welfare technologies to protect older people from danger and provide safety and security (p. 17).

But the social alarm is more than just safety. It is also about *being there* for the older person:

*Everyone can push an alarm and then someone comes. I would also like that sometimes. You push a button, and someone shows up. Sometimes they [the residents] push the button just to chat for a while. That is also a need. It doesn’t have to be about going to the bathroom or getting help with something specific. Sometimes they just want to talk for a while.*

*IP 22, female nurse*

Here the nurse is talking about the importance of relationships and socialising with others. This is something she feels the residents both want and need. ‘Pushing the button’, in this sense, seems to improve the nurse’s capacity for having and building relations with the residents. This nurse mentions that she would also like a button to push so that someone could show up for her when she has need! Though it was said as very tongue in cheek, this might also be interpreted as an admission of sometimes feeling lonely at work.

Nurses are made available through the social alarm, not only to protect but also to socialise and talk with the residents. After the Covid-19 pandemic, many residents spent a lot of time in their own apartments, rather than in the communal areas. While this was vital during the pandemic, to decrease the spread of infection, after the pandemic it meant that some residents perhaps were more lonely than necessary. Loneliness is a significant health risk for older people. The availability of staff has been shown to be vital to combat this loneliness (Gray and Worlledge, 2018). Some welfare technologies such as robot pets have also been shown to have the potential both to detect and alleviate loneliness (Latikka et al., 2021). While communal activities such as worship services, reading the newspaper aloud and more, were arranged daily in the nursing home, many residents at the unit I observed spent a lot of time in between these activities in their rooms. Pushing the button of the social alarm thus became a way of connecting with nurses and getting time to socialise a bit. Here, we can see that another form of togetherness is supported and valued by doing care with the social alarm, that of being connected and together with nurses.

### Being too available?

When reading my empirical material against the grain – against the dominant narrative – a darker side started to become visible. Being constantly available, and just a push of a button away, came with its own adverse effects. One such effect was the interruptions in other work tasks. When the nurses got an alarm signal through their mobile phone, this had to take priority, meaning that they needed to interrupt whatever they were doing to check their phone to see if the alarm came from one of ‘their’ residents and if the alarm had been answered by someone or if they had to take it. In interviews, many nurses talked about how the constant interruptions made it difficult for them to do tasks which tended to take a bit more time and needed a bit more focus such as documenting or scheduling:

*We don’t have any specific time scheduled for documentation [. . .] often when you sit down in front of the computer you get an alarm and then you need to tend to it.*

*IP 29, female nurse*

*I think it is easier to do my schedule at home because then I don’t get interrupted, and I mean it is your life for the coming five weeks you are planning. . . If you are doing it at work, you often get interrupted by colleagues or the alarm.*

*IP 32, female nurse*

These nurses both expressed a sense of stress and frustration in light of these interruptions. One might interpret this as the nurses seeing the residents as demanding and difficult^3^ when they interrupt the nurses. However, that is not my analysis. Certainly, the nurses did feel stressed when they were interrupted, but this had more to do with a feeling of there not being enough nurses to cover for each other – in other words, this was a staffing issue, not a problem with the residents. In interviews and in informal conversations, the nurses expressed a frustration towards the constant staff shortage and budget cuts.

In fact, the interruptions were stressful for nurses and residents alike. This was made worse by the fact that the nurses sometimes had to be available on another unit’s alarm because they often had one of their own residents staying there. Consequently, they regularly got around 30 alarms during a morning shift that they could not answer. Still, every time their telephone beeped, they had to check if it was one of their residents calling or not. Several nurses framed this as a work environment problem:

*This is a work environment problem because this is stressful and it’s hard. You’re in someone’s apartment who is maybe very anxious and who is having a bad morning and the only thing that happens is beeps and pings and the resident gets even more stressed and feels even worse, but there’s nothing we can do about it*

*IP 23, female nurse*

This nurse describes a situation in which she simultaneously has to check her telephone and reassure the anxious resident – not showing her own feeling of stress but instead expressing a calm and reassuring manner. Managing the expression of one’s own behaviour and feelings when working is a form of emotional labor (Hochschild, 1983). The need to suppress feelings has been found to increase stress at work (Mann and Cowburn, 2005).

So, though good care, in terms of nurse availability, was supported by the social alarm, this practice also has some serious negative consequences. The nurses may be available to residents, but sometimes the quality of that availability is seriously diminished by having to constantly divide their attention. Not only did the alarms interrupt and cause stress, but they also hindered nurses from providing good, undivided attention and situated care due to the constant and stressful interruptions.

Also often interrupted by the alarm were ‘unplanned’ social situations where the nurses sat down together with one resident or more, either in their apartment or in the communal area, just to have a chat. This was something I saw a lot during my observations. The nurses regularly sat down to make small talk with the residents while they were, for example, waiting for dinner to be served, as many residents often came to the dining room well before dinner. More often than not, this social interaction was interrupted by an alarm call. In interviews, a nurse explained that this was not unusual:

*Often when you have the chance to sit down with a resident the alarm goes off. It can be about going to the bathroom, taking a nap or about some ailment. These are things that precede everyday sociality*

*IP 27, female nurse*

This nurse described how these moments of ‘everyday sociality’ were important to her feeling like she was doing a good job. Earlier in the interview, she described how she had a sense of guilt when she felt that she did not have time to talk to lonely residents, because other things had to be prioritised. Not having enough time made it difficult to be attentive and to care for the residents in the way she wanted to.

Therefore, while the social alarm supported and helped enacting care construed as immediate nurse availability and togetherness, it also hindered this other form of unplanned and everyday togetherness. In other words, care as togetherness with the alarm excludes everyday togetherness.

### Not pushing the button

In the field, I also noticed that care as availability with social alarms came with certain conditions. One of the basic conditions, was that the residents be both willing and able to use the social alarm in the first place, something which was not the case for all residents. I was made aware of this through the disconcertment I felt in field when one of the residents was banging a drinking glass on the tray of her automated wheelchair. When I first heard the banging, I asked the nurse I was following if the resident was angry or if something had happened. ‘She does that when she wants the staff’s attention. Some of them do that.’ I was told. I felt confused. Why did the residents bang drinking glasses when they could just use the alarm? Later, when following nurse Linda again I learned more about the banging:

*Linda and I go to Ellen’s apartment. Her door is open, unlike the other apartment doors in the unit. A sign on the door reads “IMPORTANT: Keep door open between breakfast and dinner”. Linda tells me that Ellen is almost a hundred years old and spends most of her days in bed. Her social alarm is not in the form of a clock or a pedant but is instead mounted on her bedside table. She also has a small plastic lever installed above the alarm. The plastic lever is meant to help her to push the button of the alarm. The button can be a bit tricky to push, Linda explains, and if one has weak fingers a little help is useful. However, Linda goes on, Ellen rarely uses the alarm as she still finds it physically difficult to push the button. Instead, she either taps a spoon or bangs a drinking glass against her bedside table. To hear her tapping or banging, Linda says, the door must always be open during the day.*

If the dominant narrative is that the social alarm easily helps providing good and secure care through availability, the example above tells another story. This is a story about fingers not strong enough even to push a button, of new, inventive ways of getting attention and how a closed door hinders this inventive, if desperate, way of being heard. Consequently, my certainty about the use of social alarms was yet more disrupted after meeting Ellen as it made me aware that not everyone who is prescribed the social alarm might be able to use it. Some, like the resident who was banging the drinking glass against the wheelchair tray, was able to use the alarm but just thought it was easier to bang the glass. Others, like Ellen did not have the physical ability to use the alarm, despite workarounds such as the plastic lever.

A third group of residents who did not use the alarm can also be identified. These were the residents who were able to use the alarm, but who were reluctant to do so. To illustrate this, I will again turn to Linda:

*Linda tells me that Birgitta, one of the residents, has a doctor’s appointment at 10am and that Linda has told her to call her on the alarm to help her downstairs to take a taxi to the hospital. Its 9.30 and Linda is supposed to take a break soon, but she wants to be on call for Birgitta. Linda explains that Birgitta does not like to use the alarm because she feels like she is a nuisance when she does. She always talks to the staff well in advance when she knows that she will need help during the day to explain that she will call them on the alarm. At 9.35 her mobile is beeping. Birgitta is calling.*

The residents reluctant to use the alarm were predominantly women. The alarm thus excludes (some) female users as it prescribes that users have to be ‘bold’ to ‘press the button’ in the first place. While Birgitta shows such ‘boldness’ in this instance, albeit with some reluctance, others may not. This issue goes beyond the sheer functionality of the social alarm, and points to the exclusion of some users as a design bias embedded in this welfare technology.

Both this example and the example above show how care given with the social alarm, in the process of supporting some values, lives and phenomena, will necessarily exclude others (Martin et al., 2015). In this case, those who could or would not use the alarm were effectively excluded from the good and available care. While Ellen and some of the other residents had been inventive and found an of analogue alternative to the digital alarm, banging a cup or spoon, this system was fragile as it depended on the openness of doors and staff that knew that a light tap with a spoon on a bedside table could mean that someone needed help. In addition, no alternative system was in place (nor in sight) for those who were not comfortable using the alarm. For all these groups, the staff were not as readily available as for those who were physically able and comfortable using the alarm.

## Concluding discussion

In this article, I set out to show the importance of attending to ways of giving good care with welfare technology while also recognising the adverse effects that sometimes come with these care practices. I did so by asking what is valued when doing care with welfare technology and what is neglected in this process. The analysis revealed that independence, safety and some forms of togetherness and availability were valued when giving care with technology. The social alarm was key in providing immediate attentive care, situated and flexible care for the residents and helped nurses in *being there* for the residents. The analysis also showed that other forms of togetherness and availability, a stress-free working environment and functionality were values neglected in these care practices. The social alarm hindered residents from eating together with others in the dinner hall, gave rise to a lot of stress for staffs and residents alike with its constant beeping and was excluding in its functionality as not all residents could or wanted to use the alarm. Care made with the alarm also made some kinds of availability more difficult by dividing the nurses’ attention and putting extra emotional strain on them in hiding the constant distractions. If the residents were asking for social attention, someone to talk to, this is asking for the kind of undivided attention that the alarms were constantly disrupting.

Previous research shows that different forms of technology are compatible with, and even embedded in, good care (Mol et al., 2010; Van Hout et al., 2015). This is corroborated by the findings of this article. Other research has shown that the introduction of welfare technology in eldercare tends to lead to more work, new work processes and new work conditions (Ertner, 2019; Frennert, 2019; Nickelsen, 2019; Tøndel and Seibt 2019) which can all be seen as unwanted and unanticipated effects. This article similarly shows that the introduction of welfare technology has effects on the work environment. Therefore, this article underscores the importance of attending to and articulating how care *in practice* is made with technology as such attention to good care can help strengthening these practices (Mol et al., 2010). However, and in line with a *double vision of care* (Lindén & Lydahl, 2021), the article highlights the importance of being attentive to the exclusions reproduced in and through care practices. This is so, especially in times when the promises regarding the benefits of welfare technologies are increasing. This approach keeps both techno-optimism and techno-phobia at bay.

By utilising a double vision, it is possible to identify and analyse both the goods and the bads consequent from use of such technologies. Inspired by a care-in-practice approach, the goods – which are often implicit for practitioners themselves – can be opened up and discussed, and lessons learnt can be transported from one practice to another (Pols, 2008). Meanwhile, the exclusions can also be highlighted, and a discussion can be held about whether it is possible to keep the goods while finding ways of tinkering with, or working around, adverse effects – or indeed, if the adverse effects are so serious that one should ponder the withdrawal of the technology altogether. Perhaps such a discussion will find that it is not the technology *per se* that is a problem, but rather staff shortages or overall working conditions.

A methodological limitation of the study is its somewhat small scope. However, the theoretical implications about good care made with technology discovered here, and its adverse effects, can still instruct researchers, practitioners and policymakers alike regarding important lessons about care made with welfare technology. More importantly, as (author and author) have shown, a double vision of care is a perspective which has much to offer health and society beyond the context of eldercare and welfare technology since such care can be found in a variety of locations.

## Notes

1. While the concept of welfare technology is rather new, technologies reminiscent of welfare technologies, such as early versions of the social alarm, have been used in eldercare services in Sweden since the 1970s. These versions of the social alarm consisted of a wall-mounted apparatus, but were soon replaced by portable systems connected to the telephone network. Until the 1990s, social alarms were connected to the telephone system, and the alarm systems were run by the municipality and Televerket, a Swedish State authority acting as a state-owned corporation (public enterprise), responsible for telecommunications in Sweden from 1853 until 1993.

2. At the nursing home, this sense of safety and security is even part of the social alarm’s name. It is called *The Safety Alarm* (Trygghetslarmet, in Swedish).

3. Medical sociologists have theorised that particular patients in various health care settings are labelled as difficult and demanding by health professionals (Groves, 1978; Koekkoek et al., 2011), and failing to comply with their ‘sick role’ (Parsons, 1951).
